# Prognostication in Epilepsy with Integrated Analysis of Blood Parameters and Clinical Data

**DOI:** 10.3390/jcm13185517

**Published:** 2024-09-18

**Authors:** Kyung-Il Park, Sungeun Hwang, Hyoshin Son, Jangsup Moon, Soon-Tae Lee, Keun-Hwa Jung, Ki-Young Jung, Kon Chu, Sang Kun Lee

**Affiliations:** 1 Department of Neurology, Seoul National University College of Medicine, Seoul 03080, Republic of Korea; ideopki@gmail.com (K.-I.P.); jangsup.moon@gmail.com (J.M.); staelee@snu.ac.kr (S.-T.L.); jungkh@gmail.com (K.-H.J.); jungky@snu.ac.kr (K.-Y.J.); stemgen1@snu.ac.kr (K.C.); 2 Department of Neurology, Seoul National University Healthcare System Gangnam Center, Seoul 06236, Republic of Korea; 3 Department of Neurology, Ewha Womans University Mokdong Hospital, Seoul 07985, Republic of Korea; neurosung@gmail.com; 4 Department of Neurology, Catholic University of Korea Eunpyeong St Mary’s Hospital, Seoul 03312, Republic of Korea; hson727@gmail.com; 5 Department of Genomic Medicine, Seoul National University Hospital, Seoul 03080, Republic of Korea; 6 Department of Neurology, Seoul National University Hospital, Seoul 03080, Republic of Korea

**Keywords:** epilepsy, outcome, prediction, blood

## Abstract

**Background/Objectives**: Determining the outcome of epilepsy is crucial for making proactive and timely treatment decisions and for counseling patients. Recent research efforts have focused on using various imaging techniques and EEG for prognostication; however, there is insufficient evidence regarding the role of blood parameters. Our study aimed to investigate the additional prognostic value of routine blood parameters in predicting epilepsy outcomes. **Methods**: We analyzed data from 1782 patients who underwent routine blood tests within 90 days of their first visit and had a minimum follow-up duration of three years. The etiological types were structural (35.1%), genetic (14.2%), immune (4.7%), infectious (2.9%), and unknown (42.6%). The outcome was defined as the presence of seizures in the last year. **Results**: Initially, a multivariate analysis was conducted based on clinical variables, MRI data, and EEG data. This analysis revealed that sex, age of onset, referred cases, epileptiform discharge, structural etiology, and the number of antiseizure medications were related to the outcome, with an area under the curve (AUC) of 0.705. Among the blood parameters, fibrinogen, bilirubin, uric acid, and aPTT were significant, with AUCs of 0.602, 0.597, 0.455, and 0.549, respectively. Including these blood parameters in the analysis slightly improved the AUC to 0.710. **Conclusions**: Some blood parameters were found to be related to the final outcome, potentially paving the way to understanding the mechanisms of epileptogenesis and drug resistance.

## 1. Introduction

Despite dedicated medical treatment, approximately 30% of epilepsy patients suffer from recurrent seizures. Among medically intractable epilepsy patients, approximately half are surgically remediable. Given that a longer duration of epilepsy leads to poorer surgical outcome [1,2], the prediction of the final outcome of medical treatment could save time until surgical resection and could also help in selecting candidates for novel treatment without delay [3], as well as assisting in patient counseling. Additionally, a misdiagnosis or delayed prediction of the outcome of epilepsy can have significant losses, in terms of healthcare cost and ensuing social stigma, so an appropriate diagnosis is of paramount importance, and blood markers can help with this.

According to numerous previous articles, clinical factors related to drug resistance include onset age [4,5,6], the number of seizures [7], the response of initial antiseizure medication (ASM) [8], the presence of febrile seizures [5,9], and epilepsy duration [10,11]. Electroencephalography (EEG) biomarkers, such as epileptiform discharges [12], are used to predict outcomes. Image factors include the presence of hippocampal sclerosis [4] and any structural etiology [13]. Liquid biomarkers have also been investigated; for example, serum High-mobility group box 1 (HMGB1) and interleukin (IL)-1β have been related to epilepsy outcomes in the pediatric population [14], and neuron-specific enolase [15] has been used to predict the outcome. However, there is a paucity of large-scale human studies investigating blood parameters from routine clinical settings in epilepsy.

In this study, we aimed to focus on routine blood parameters in relation to the final outcomes in a relatively large-sized population from a single center.

## 2. Materials and Methods

### 2.1. The Study Population

A total of 2586 patients from SERENADE (Seoul national university hospital adult Epilepsy Retrospective cohort in the Era of Newer Antiseizure Drug Exposure), with clinical information, EEG data, image data, and routine blood tests, were evaluated. The patients met the following criteria: (1) visited our epileptologists’ clinic from January 2008 to January 2018 for the first time; (2) diagnosed with epilepsy with the International League Against Epilepsy (ILAE) definition; (3) prescribed any ASM for more than three months; and (4) had at least a three-year or more follow-up period. This study was approved by the Seoul National University Hospital Institutional Review Board (2308-010-1455) and followed the principles of the Declaration of Helsinki. The requirement for written consent was waived due to the retrospective design.

### 2.2. Collected Data Characteristics

The patients’ lists and laboratory results were extracted from the clinical database warehouse in our institution. Additionally, investigators reviewed electronic medical records thoroughly, including sex, onset age, number of seizures before ASM initiation, newly diagnosed vs. referred case, seizure classification, epilepsy classification, etiology, history of febrile convulsion, family history of epilepsy, and epilepsy surgery history. Data from routine blood tests—including white blood cells (WBCs), hemoglobin, platelets, neutrophils, lymphocytes, monocytes, eosinophils, basophils, absolute neutrophil counts (ANCs), MCV, MCH, MCHC, RDW, protein, albumin, bilirubin, aspartate aminotransferase, alanine aminotransferase, alkaline phosphatase, calcium, phosphorus, glucose, blood urea nitrogen, creatinine, uric acid, Na/K/Cl, tCO2, prothrombin time, activated partial thromboplastin time (aPTT), fibrinogen, and total cholesterol—were collected only in cases sampled within 90 days of the first visit. The routine blood test was performed at an outpatient clinic or on admission, according to the individual situation in the real-world scenario.

Magnetic resonance imaging (MRI) lesions were defined as the most relevant lesion in the patient’s epilepsy, based solely on MRI through a consensus of three experienced epileptologists (KIP, SH, and SKL). In surgical cases, the pathologic diagnosis takes priority when there is a discrepancy between the pathologic and radiologic diagnoses. EEG findings were defined on the first test during the entire follow-up duration, irrespective of the routine or video-EEG. The epileptic waveforms include periodic discharge, rhythmic discharge, and rhythmic or isolated spike/wave. The final outcome was defined by seizures that occurred during the last year of follow-up, enabling classification as seizure-free or seizure-persistent. In surgical cases, the outcome was assessed based on the most recent year before the surgery.

### 2.3. Statistical Analysis

The numerical values are expressed as numbers or the mean ± standard deviation. Student’s *t*-test was used for continuous variables, and the Mann–Whitney test was used for non-continuous variables. A Chi-square test was also performed. To ensure the independent factor status of the final outcome, we performed multiple linear regressions, which included both variables that showed significance in univariate analysis and potentially meaningful variables. Additionally, we performed multiple comparisons using a false discovery rate of 0.05. To compare the mean values across the three groups, we conducted an analysis of variance (ANOVA), followed by post hoc analysis. The statistical significance was set as a two-tailed *p*-value < 0.05. SPSS (version 25, IBM, Chicago, IL, USA) or GraphPad Prism (version 9, Dogmatics, San Diego, CA, USA) was used for all statistical analyses.

## 3. Results

This cohort comprised the total population of patients who visited our epilepsy center for the first time over a ten-year period from 2008 to 2017. Of the 2586 patients in the entire cohort, 1782 patients (45.7% female) who had routine blood test results within three months of the initial clinic visit were finally analyzed. Comparing the final outcome between the presence and absence of the initial blood test, we found that there was no significant difference in outcome (*p* = 0.676).

The gap between the first visit and epilepsy onset was 6.9 ± 9.6 years (range, 0–65). Among the patients, newly diagnosed patients were the population who had no history of epilepsy treatment or were managed for less than six months following their clinic visit, if any, at our center. The proportion of newly diagnosed patients was 52.1%. The onset age of epilepsy was 29.6 ± 19.6 years, and the age of the first visit to our center was 37.5 ± 18.0 years. Following the 2017 ILAE classification, the number of cases of focal epilepsy, generalized epilepsy, combined, and unknown was 1387 (77.8%), 275 (15.4%), 98 (5.5%), and 22 (1.2%), respectively. Regarding etiology, structural etiology (626, 35.1%) was the most common. Genetic (253, 14.2%), immune (84, 4.7%), infectious (51, 2.9%), hypoxic (5, 0.3%), and metabolic (3, 0.1%) etiologies followed, in order of frequency. However, 760 patients (42.6%) had an unknown etiology (Table 1). EEG data, routine or long-term, were available in 1631 patients (91.5%). MRI under an epilepsy-specific protocol in our institution was obtained in 1553 (87.1%) patients. Surgical intervention, including resective operation and vagal nerve stimulation, was performed in 58 patients (3.3%).

The final seizure freedom rate was 65.8%, representing 1173 patients. We sought to identify independent determinants of the final outcome using initial clinical status, EEG data, and MRI data. Multivariate analysis revealed that six clinical parameters were significantly associated with the final outcome: sex (*p* = 0.005), epilepsy onset age (*p* = 0.011), referred cases (*p* = 0.007), epileptiform discharges on EEG (*p* = 0.002), the number of ASMs (*p* < 0.001), and structural etiology (*p* = 0.021) (Table 2).

Based on multiple logistic regression analysis, we developed the following equation to predict the final outcome:

Equation (1) = (−0.373 × sex) + (0.445 × epileptiform discharge) + (0.387 × structural etiology) + (0.390 × number of initial ASM) − (0.021 × onset age) + (0.456 × referred cases).

In this model, male sex, the presence of epileptiform discharges, structural etiology, and referred cases are coded as 1. The area under the curve (AUC) for this model was 0.705 (*p* < 0.001).

**Figure 1:**
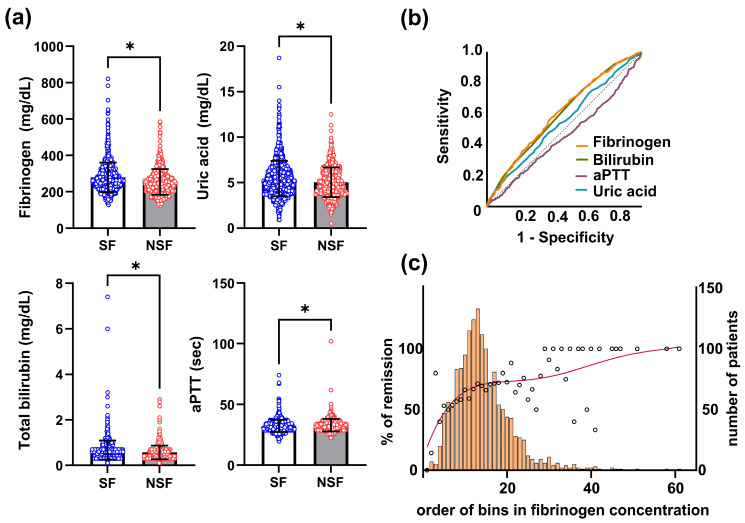
Routine blood markers related to the final epilepsy outcome. (**a**) The final outcome showed a significant correlation with the blood level of fibrinogen, uric acid, total bilirubin, and activated partial thromboplastin. (**b**) Fibrinogen levels showed the highest area under the curve (AUC) among statistically significant blood parameters on the final outcome. The dotted line represents random chance, indicating an AUC of 0.5. (**c**) The distribution of fibrinogen levels in relation to the final outcome is illustrated, along with the corresponding number of patients in each bin. Bars indicate the number of patients in each bin, while circles represent the percentage of remission among the patients in the bins. SF, seizure-free; NSF, nonseizure-free; aPTT, activated partial thromboplastin. * indicates statistical significance.

In the next step, we conducted a similar analysis, incorporating routine blood parameters. Multiple logistic regression identified epileptiform discharges (*p* = 0.001), structural etiology (*p* = 0.005), the number of initial ASMs (*p* < 0.001), onset age (*p* = 0.038), referred cases (*p* = 0.011), fibrinogen (*p* = 0.002), uric acid (*p* = 0.016), aPTT (*p* = 0.003), and bilirubin (*p* = 0.045) as independent prognostic factors (Table 3) (Figure 1a).

The following equation was developed based on this analysis: Equation (2) = (0.510 × Epileptiform discharge) + (0.525 × Structural etiology) + (0.349 × Number of initial ASM) − (0.019 × Onset age) + (0.472 × referred case) − (0.441 × bilirubin) − (0.117 × uric acid) + (0.048 × aPTT) − (0.004 × fibrinogen). The AUC was 0.710 (*p* < 0.001), which was slightly improved compared to Equation (1), not considering the blood parameters. The AUCs for fibrinogen, bilirubin, uric acid, and aPTT were 0.602, 0.597, 0.549, and 0.455, respectively (Figure 1b). Using a cutoff that maximizes the value of sensitivity–specificity + 1, the specificity, sensitivity, positive predictive value, and negative predictive value were 0.58, 0.73, 0.53, and 0.77 in Equation (1) and 0.59, 0.71, 0.52, and 0.77 in Equation (2), respectively. An outcome comparison according to the meaningful parameters is summarized in Table 4.

Focusing on blood fibrinogen levels, which had the highest AUC among the blood parameters, we found that fibrinogen levels were significantly higher in the seizure-free group compared to the seizure-persistent group (Figure 1a,c). Patients in the high-quartile group for fibrinogen levels had a better final outcome than those in the low-quartile group (*p* < 0.01).

**Figure 2:**
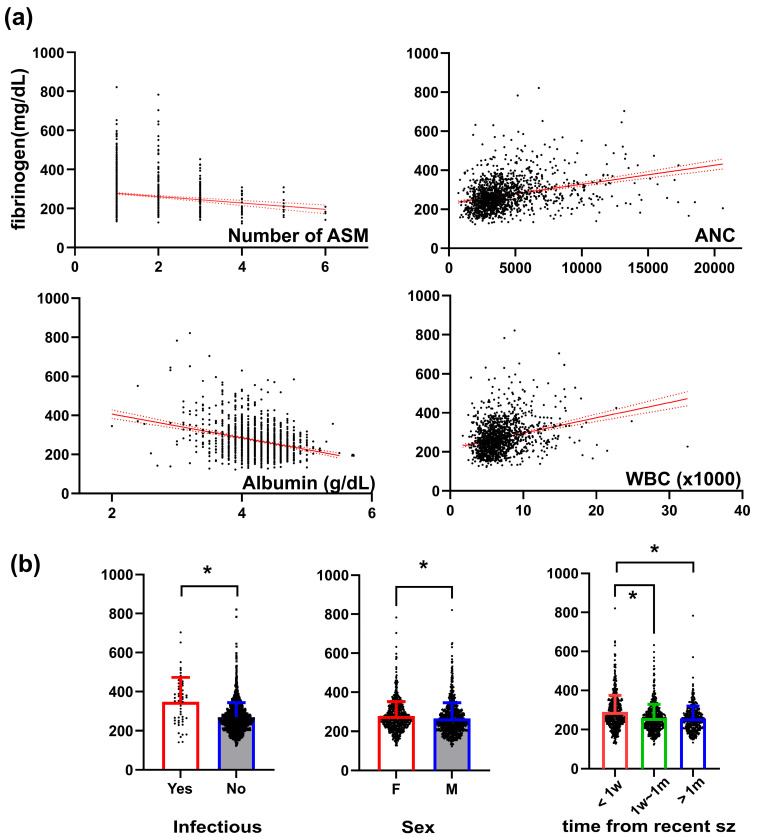
Association of factors with initial level of blood fibrinogen. (**a**) Correlation of fibrinogen level with meaningful continuous variables. (**b**) Comparison of fibrinogen with meaningful categorical variables. Sz, seizure; ANC, absolute neutrophil count; WBC, white blood cell. * indicates statistical significance.

Multiple linear regression with the fibrinogen level as the dependent variable showed that the final outcome, sex, infectious etiology, WBC, ANC, albumin, and the number of initial ASMs were significantly associated with fibrinogen levels, after adjusting for multiple comparisons (Table S1). The correlations between fibrinogen levels and associated factors are illustrated in Figure 2a. Female sex, structural or infectious etiology, and recent seizure were associated with higher fibrinogen levels (Figure 2b).

## 4. Discussion

In this study, we found that several blood parameters measured within three months after the first visit to our center were associated with the final outcome of epilepsy in a large cohort. Considering the routine blood parameters, the prognostication is slightly improved compared to when analyzing only clinical variables, MRI data, and EEG data.

Among blood parameters, we found that fibrinogen, uric acid, bilirubin, and aPTT were associated with the final outcome. Among these, we focused on blood fibrinogen, which is a novel parameter and had the highest AUC value. Blood fibrinogen is produced by the liver and plays a role in inflammatory and coagulation cascades in a wide spectrum of neurological diseases, including stroke, spinal cord injury, brain trauma, multiple sclerosis, and Alzheimer’s disease [16]. In acute traumatic brain injury [17], fibrinogen in blood extravasates into the brain parenchyma across disrupted blood–brain barriers and interacts with brain cells, producing reactive oxidative species and inflammatory cytokines such as IL-6 and C-C motif chemokine ligand 2 (CCL2), and induced IL-6 further amplifies fibrinogen, finally leading to neuro-inflammatory cascades [18,19]. Penetrating fibrinogen after traumatic brain injury also activates microglia and astroglia, leading to subsequent cell damage [20].

Several previous clinical studies demonstrated an apparently harmful association, showing high fibrinogen and low attention/executive function in mild cognitive impairment [21] and progression to dementia [22]. Since fibrinogen has been regarded as a systemic inflammatory marker, the status of hyperfibrinogenemia as a poor prognostic factor was explained by its inflammatory action. In epilepsy, ample evidence has accumulated that systemic inflammation crosstalk with brain-borne inflammation leads to epileptogenesis. Patients with a higher seizure burden showed higher levels of blood markers of inflammation, such as C-reactive protein and IL-6 [23]. However, we found that a high fibrinogen level was associated with a good final outcome in this study.

Neuro-inflammatory processes can be diverse, and systemic immune reactions are more complex in the epilepsy process. The generally accepted concept is that systemic immunity aggravates epilepsy, which has been proven in animal and clinical studies. Blocking leukocyte–endothelial adhesion prevents epilepsy in animal models [24]. An animal study also supported that the reduction in recruiting circulating immune cells decreased brain-borne neuro-inflammation and neuronal damage using the chemokine receptor 2 knockout model [25]. The results from our prior clinical study also indicated an immediate increase in serum IL-6 concentration following generalized tonic–clonic seizures [26], supporting the established concept of a positive feedback loop between seizures and systemic inflammation. However, another study contradicted this finding, where T-cell/B-cell-depleted mice showed exacerbated seizures following the use of kainic acid [27]. This contradiction might be due to different models of epilepsy and, more importantly, different time points during the course of epilepsy. The neuro-inflammatory role may differ between immediately after a seizure and during the interseizure baseline state. It needs to be considered that the pro-inflammatory process can be balanced with the anti-inflammatory process.

Although the mechanism is unclearly explained, data supporting that high fibrinogen levels are a factor indicative of good prognosis in neurological disease also exist. Low fibrinogen concentration after traumatic injury was associated with an unfavorable prognosis [28,29]. A fibrinogen level < 2.0 g/L is an independent prognostic factor for 3-month mortality. By contrast, a level > 3 g/L is an independent prognostic factor for favorable outcomes at 3 months for traumatic brain injury. Another study showed a positive association of blood fibrinogen in patients with amyotrophic lateral sclerosis [30]. In this study, a high level of blood γ-fibrinogen, which accounts for ~8% of plasma fibrinogen, was associated with a better disease outcome. Although that paper suggested an alternative microglial activation, called M2, as a possible mechanism, the protective mechanism of fibrinogen is still obscure. The plasma fibrinogen level was lower in idiopathic generalized epilepsy [31] than in controls, which is consistent with our results in part despite the small number of patients included, implying that seizures are related to low plasma fibrinogen. However, this study did not show a difference when comparing seizure-free and not, possibly due to the small study population.

Fibrinogen acts both on thrombosis and neuro-inflammation. This study showed that fibrinogen was independently associated with the number of WBCs and ANC, which are known as immune and inflammatory markers. Therefore, inflammatory mechanisms, rather than thrombosis, seem to be more attributed to the epilepsy mechanism. Alongside inflammatory reactions, fibrinolysis occurs in epilepsy [32]. We can speculate that seizures themselves induce fibrinolysis, akin to traumatic brain injury. This speculation is supported by the finding that a shorter interval between sampling and recent seizures is associated with a higher fibrinogen level (Table S1), although significance did not reach the statistical threshold after applying a false discovery rate of 0.05.

A statistical relationship between the blood level of fibrinogen and the infectious etiology of epilepsy suggests a plausible direct influence of the infectious cause on fibrinogen levels. However, it is essential to emphasize that the association between fibrinogen levels and a good final outcome was not a result of infection because the statistics revealed that neither infectious nor immune etiology had a significant relationship with the final outcome. Interestingly, the fibrinogen level was associated with the number of ASMs. The lower the number of ASMs was, the higher the fibrinogen level. From this finding, we speculate that ASM can influence systemic inflammatory markers or, more specifically, fibrinogen levels. The findings presented in previous papers also support this assumption [33,34,35,36]. Certain ASMs might play an anti-inflammatory role, which is evidenced by low fibrinogen. 

A shorter aPTT is associated with a better outcome in epilepsy. The mechanism by which aPTT influences epilepsy outcomes is not yet understood. However, a shorter aPTT, indicating an increased intrinsic pathway, can produce more fibrinogen with higher turnover in the coagulation pathway. How this independently links to the outcome needs to be clarified in further studies.

A high level of uric acid is also associated with a better outcome in epilepsy. A previous study involving 5672 epilepsy patients demonstrated that the hyperuricemic group, prior to their epilepsy diagnosis, had a lower conversion rate to drug-resistant epilepsy [37]. Although our final outcome was dichotomized into seizure-free or not, and our population included referred patients from other hospitals, the association between uric acid levels and seizure outcomes is consistent with previous findings. This paper suggested that the protective role of uric acid is due to its antioxidant effect, which counteracts seizure-induced oxidative stress. Elevated serum uric acid levels are often related to the excessive consumption of red meat. The habit of consuming a high-protein diet, including excessive meat intake, might contribute to the protective effect against seizures, similar to the effects of a ketogenic diet.

We also found that a high serum level of bilirubin was associated with a good outcome. A previous animal study with a piglet model of seizure demonstrated that bilirubin treatment before and after seizure induction significantly decreased the reactive oxygen species production in cerebral vessels and astrocytes, highlighting the role of the antioxidant effect of bilirubin [38].

This study had several limitations. First, blood samples were taken at various time points following a recent seizure. As previously mentioned, it is imperative to consider that acute seizures can influence blood parameters. Thus, we included this covariate in the statistical analysis. Second, we did not assess other acute inflammatory markers, such as C-reactive protein or interleukins. It is possible that, even in cases with high levels of fibrinogen without infectious or immune etiology, there may exist a specific subgroup of patients in whom the inflammatory response plays a more prominent role in epilepsy. Further research using multiple markers of inflammation in more refined populations is needed to clarify this aspect. Third, this study only included subjects who underwent blood sampling within three months after their first visit. This three-month period aligns with our clinical setting, where blood tests are typically performed at admission after the first outpatient visit, usually between one and three months. This approach may have introduced selection bias by not including the entire population of epilepsy patients. However, the final outcome did not differ based on the presence or absence of the initial blood test.

## 5. Conclusions

While the mechanisms of all blood parameters influencing epilepsy outcomes were not clearly explained, we attempted to incorporate routine blood parameters to predict the epilepsy outcome in a large population from a single institution. Although the predictive power is not drastically improved by incorporating blood parameters, this approach enables us to gain deeper insights into the mechanistic role of blood markers in epilepsy. More studies in drug-naïve patients, observations of longitudinal changes, and external validation are necessary to verify our results.

## Figures and Tables

**Table 1:** Demographics of study population.

Clinical Parameters	Number of Patients (%)
Sex (Female/Male)	814:968 (45.7%:54.3%)
Onset age	
mean ± SD (range)	31.0 ± 20.2 (0–91)
Missing, N	32
Age of first visit (range)	37.5 ± 18.0 (6–91)
Follow-up duration, month (range)	104.5 ± 30.5 (36–161)
Epilepsy duration, year	
mean ± SD (range)	6.3 ± 9.5 (0–56)
Missing, N	32
Newly diagnosed	1026 (57.6%)
Unknown	166 (9.3%)
Seizure types	
Focal	1476 (82.9%)
Generalized	281 (15.8%)
Unknown	23 (1.3%)
Missing, N	2
Epilepsy classification	
Focal	1387 (77.8%)
Generalized	275 (15.4%)
Combined	98 (5.5%)
Unknown	22 (1.2%)
Etiology	
Structural	626 (35.1%)
Genetic	253 (14.2%)
Immune	84 (4.7%)
Infectious	51 (2.9%)
Hypoxic	5 (0.3%)
Metabolic	3 (0.2%)
Unknown	760 (42.6%)
History of febrile seizure	138 (7.7%)
Family history of epilepsy	55 (3.1%)
Presence of epileptiform discharge on EEG	
N (%)	429 (26.3%)
Missing, N	151
Lesion on MRI	
N (%)	764 (49.2%)
Missing, N	229
Hippocampal sclerosis	
N (%)	101 (6.5%)
Missing, N	229
Final seizure-free	609 (34.2%)
Number of initial ASMs	Range 1–6 (median 1)
Sampling time from recent seizure	
<1 week	608 (39.8%)
1 week~1 month	539 (35.3%)
>1 month	379 (24.8%)

SD, standard deviation; N, number of patients; ASM, antiseizure medication.

**Table 2:** Multiple logistic regression for the final outcome using clinical status, EEG, and MRI.

	B	SE	Wald	*p* Value	Exp (B)
Sex (Male = 1)	−0.373	0.132	7.990	0.005	0.688
Febrile seizure	0.407	0.240	2.879	0.090	1.502
Epileptiform discharge	0.445	0.144	9.581	0.002	1.560
MRI lesion	0.113	0.165	0.472	0.492	1.120
Focal seizure	1.179	0.663	3.162	0.075	3.250
Generalized epilepsy	0.974	0.759	1.649	0.199	2.649
Structural etiology	0.387	0.167	5.357	0.021	1.472
Genetic etiology	−0.524	0.520	1.012	0.314	0.592
Hippocampal sclerosis	0.236	0.273	0.746	0.388	1.266
Number of initial ASMs	0.390	0.098	15.949	<0.001	1.477
Age at first visit	−0.008	0.009	0.898	0.343	0.992
Onset age	−0.021	0.008	6.417	0.011	0.979
Follow-up duration	0.003	0.002	1.712	0.191	1.003
Referred cases	0.456	0.168	7.389	0.007	1.578
Constant	−2.081	0.728	8.165	0.004	0.125

SE, standardized error.

**Table 3:** Prognostic factors including blood parameters.

	B	SE	Wald	*p* Value	Exp (B)
Clinical parameters					
Sex (Male = 1)	−0.165	0.179	0.845	0.358	0.848
Febrile seizure	0.502	0.259	3.769	0.052	1.652
Epileptiform discharge	0.510	0.160	10.186	0.001	1.665
MRI lesion	0.003	0.184	0.000	0.988	1.003
Focal seizure	1.449	0.809	3.207	0.073	4.257
Generalized epilepsy	0.952	0.908	1.099	0.294	2.591
Structural etiology	0.525	0.188	7.770	0.005	1.690
Genetic etiology	−0.176	0.646	0.074	0.785	0.839
Hippocampal sclerosis	0.167	0.301	0.308	0.579	1.182
Number of initial ASMs	0.349	0.106	10.794	0.001	1.418
Age at first visit	−0.001	0.010	0.012	0.914	0.999
Onset age	−0.019	0.009	4.324	0.038	0.981
Follow-up duration	0.001	0.002	0.105	0.746	1.001
Referred cases	0.472	0.185	6.500	0.011	1.603
Blood parameters					
WBCs	−0.042	0.134	0.100	0.752	0.959
Neutrophils	0.025	0.026	0.908	0.341	1.025
Lymphocytes	0.015	0.024	0.361	0.548	1.015
ANC	0.000	0.000	0.106	0.745	1.000
Albumin	0.005	0.224	0.000	0.983	1.005
Bilirubin	−0.441	0.253	3.033	0.045	0.643
AST	0.000	0.003	0.025	0.875	1.000
Glucose	−0.003	0.002	1.082	0.298	0.997
BUN	−0.027	0.020	1.734	0.188	0.974
Creatinine	0.366	0.237	2.381	0.123	1.442
Uric acid	−0.117	0.049	5.839	0.016	0.889
aPTT	0.048	0.016	8.851	0.003	1.049
Fibrinogen	−0.004	0.001	9.402	0.002	0.996
Constant	−3.705	2.626	1.991	0.025	0.021

WBC, white blood cell; ANC, absolute neutrophil count; AST, aspartate transferase; BUN, blood urea nitrogen; aPTT, activated partial thromboplastin.

**Table 4:** Outcome comparison according to meaningful parameters.

Outcome	Nonseizure-Free (N = 609)	Seizure-Free (N = 1173)	*p* Value
Epileptiform discharge	181 (29.7%)	248 (21.1%)	<0.001
Structural etiology	249 (40.9%)	377 (32.1%)	<0.001
Number of initial ASMs			<0.001
1	386 (63.4%)	968 (82.5%)	
2	113 (18.6%)	141 (12.0%)	
3	66 (10.8%)	53 (4.5%)	
4	27 (4.4%)	10 (0.9%)	
5	13 (2.1%)	1 (0.1%)	
6	4 (0.7%)	0 (0.0%)	
Onset age	23.8 ± 16.8	34.7 ± 20.9	<0.001
Newly diagnosed	250 (41.1%)	776 (66.2%)	<0.001
Uric acid	5.0 ± 1.6	5.4 ± 1.9	<0.001
aPTT	33.6 ± 16.8	32.4 ± 5.0	0.023
Fibrinogen	254.0 ± 70.7	279.5 ± 80.7	<0.001
Bilirubin	0.6 ± 0.3	0.7 ± 0.4	<0.001

## Data Availability

The authors confirm that the data supporting the findings of this study are available on reasonable request.

## Supplementary Materials

The following supporting information can be downloaded at https://www.mdpi.com/article/10.3390/jcm13185517/s1, Table S1: Multiple linear regression for fibrinogen level.
