# Supplementary material for: Prognostication in Epilepsy with Integrated Analysis of Blood Parameters and Clinical Data
Source: J Clin Med. 2024 Sep 18;13(18):5517. doi: 10.3390/jcm13185517 (PMC11432444; doi:10.3390/jcm13185517)
Supplement: Supplementary file 1 [file jcm-13-05517-s001.zip › jcm-3196519-supplementary.pdf]

Table S1 Multiple linear regression for fibrinogen level.

	B	Standardized error	95% Confidence interval	β	Unadjusted P value
*Final outcome	-10.946	4.259	-19.3 – -2.59	-0.066	0.010*
*Sex	-10.346	3.929	-18.05 – -2.64	-0.066	0.009*
History of febrile seizure	-5.488	6.795	-18.82 – 7.84	-0.020	0.419
MRI lesion	1.523	4.632	-7.56 – 10.61	0.010	0.742
Generalized epilepsy	10.946	6.093	-1.01 – 22.9	0.046	0.073
Structural etiology	10.654	4.861	31.41 – 75.14	0.066	0.029
*Infectious etiology	53.277	11.145	-0.01 – 0.82	0.120	<0.001*
Onset age	0.405	0.213	7.24 – 20.18	0.104	0.057
*WBC	13.709	3.297	-0.5 – 2.16	0.448	<0.001*
Neutrophil	0.833	0.678	-2.36 – 0.13	0.129	0.219
Lymphocyte	-1.114	0.636	-0.02 – 0	-0.153	0.080
*ANC	-0.014	0.005	-41.48 – -19.29	-0.414	0.004*
*Albumin	-30.386	5.654	-13.76 – -4.13	-0.148	<0.001*
*Number of initial ASM	-8.942	2.453	-0.11 – 0.81	-0.095	<0.001*
Age of sampling	0.348	0.235	1.12 – 20.19	0.080	0.140
Sampling time from a recent seizure	-5.440	2.500	-10.34 – -0.54	-0.055	0.030

WBC, white blood cell; ANC, absolute neutrophil count; ASM, antiseizure medication.

* Statistically significant after multiple comparisons.
